# Supplementary figures and images for: Involvement of Glucosamine 6 Phosphate Isomerase 2 (GNPDA2) Overproduction in β-Amyloid- and Tau P301L-Driven Pathomechanisms
Source: Biomolecules. 2024 Mar 25;14(4):394. doi: 10.3390/biom14040394 (PMC11048700; doi:10.3390/biom14040394)

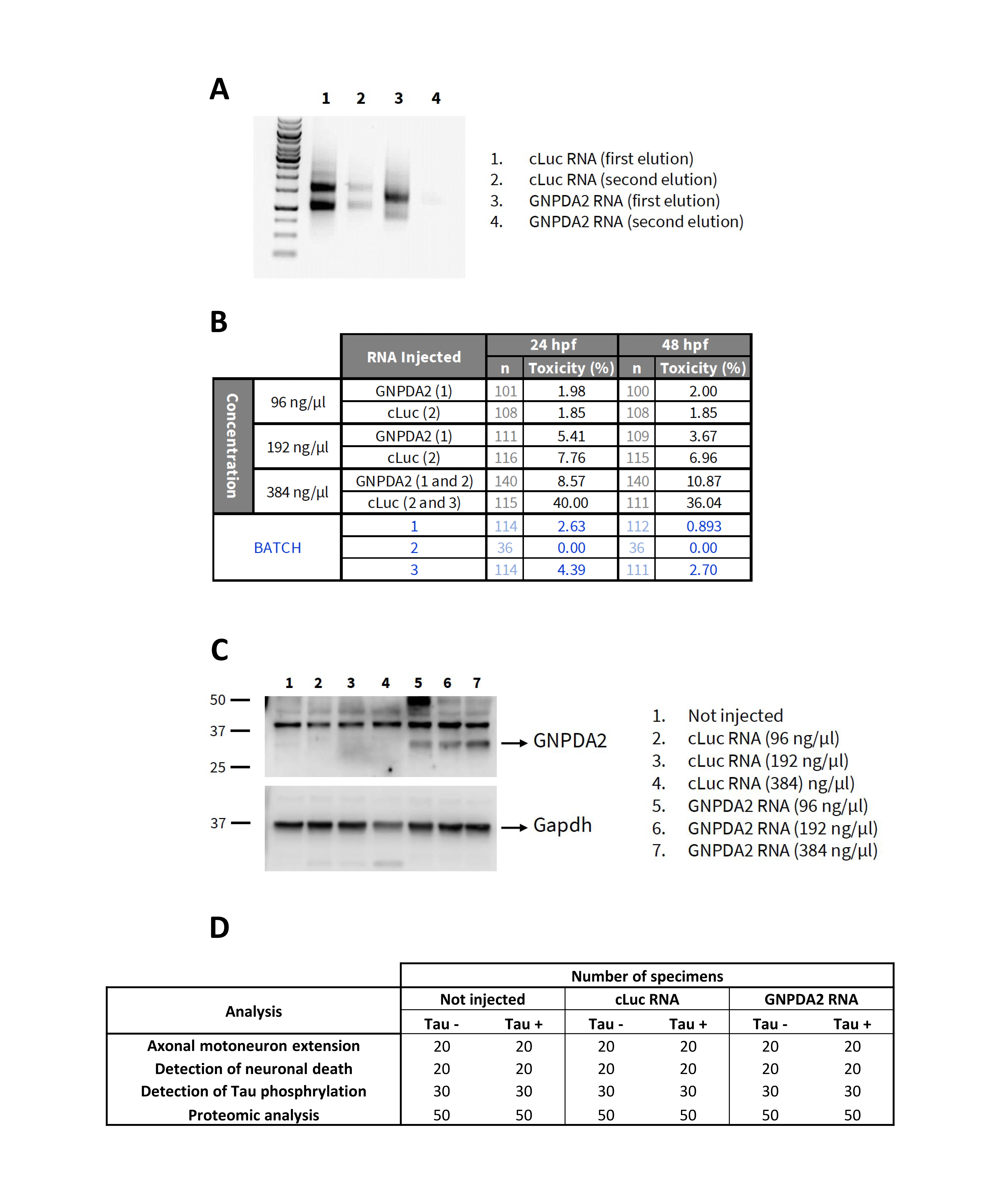

Supplement: Supplementary file 1 [file biomolecules-14-00394-s001.zip › Supplementary-Figure S1.tif]

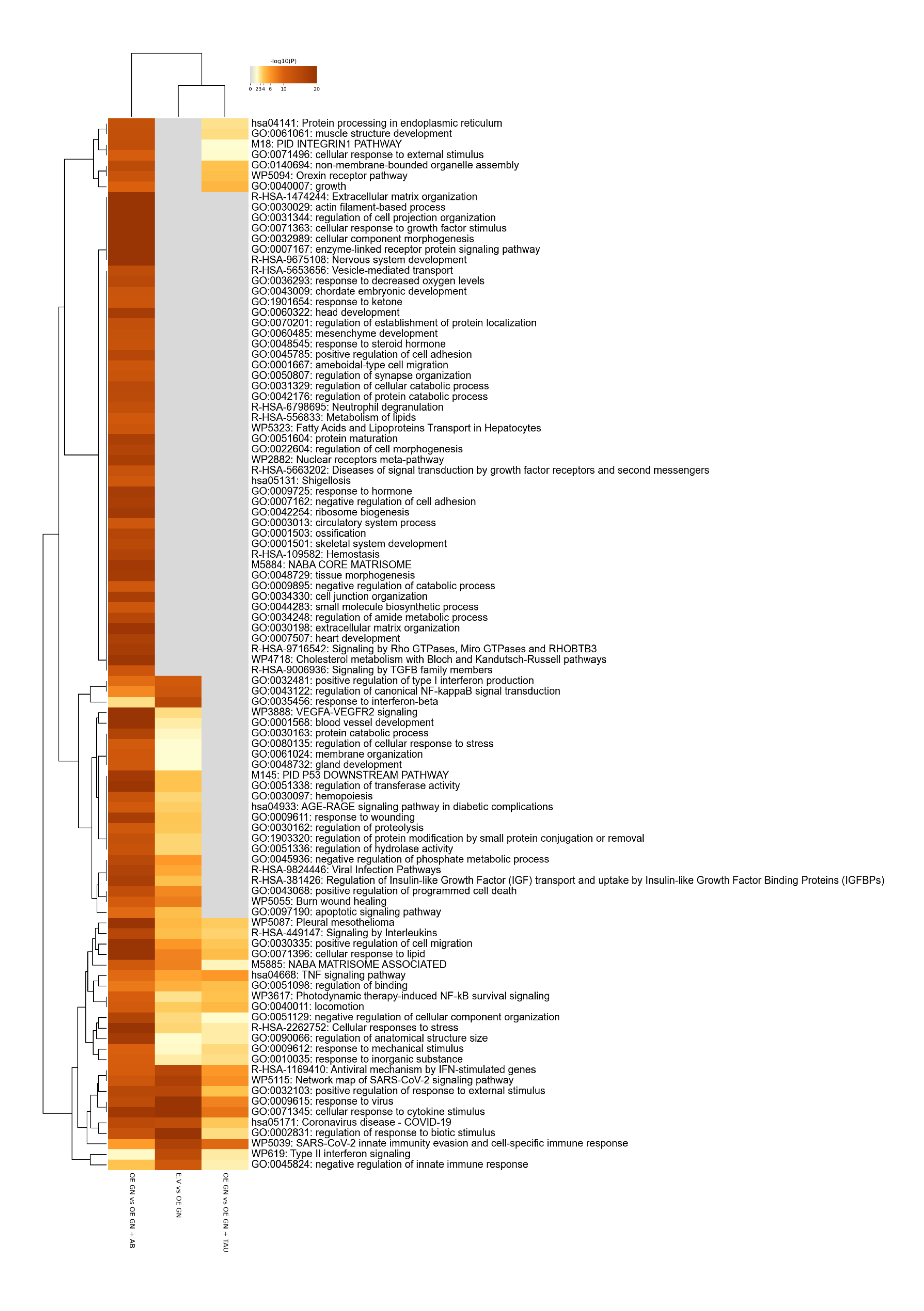

Supplement: Supplementary file 1 [file biomolecules-14-00394-s001.zip › Supplementary-Figure S2.tif]

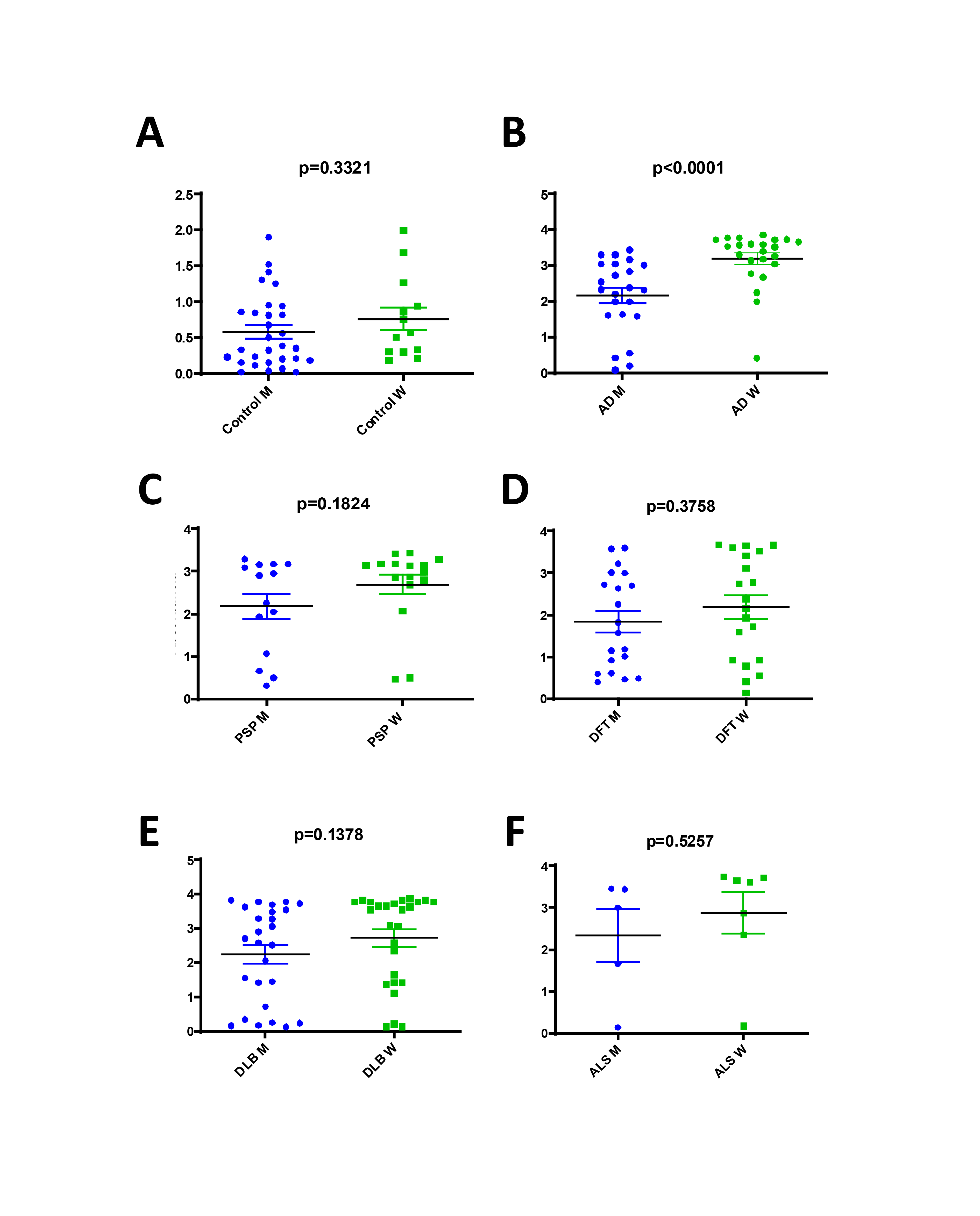

Supplement: Supplementary file 1 [file biomolecules-14-00394-s001.zip › Supplementary-Figure S3.tif]
